# An Interactive, Mobile-Based Tool for Personal Social Network Data Collection and Visualization Among a Geographically Isolated and Socioeconomically Disadvantaged Population: Early-Stage Feasibility Study With Qualitative User Feedback

**DOI:** 10.2196/resprot.6927

**Published:** 2017-06-22

**Authors:** Katherine S Eddens, Jesse M Fagan, Tom Collins

**Affiliations:** ^1^ Department of Health, Behavior & Society College of Public Health University of Kentucky Lexington, KY United States; ^2^ Department of Management Gatton College of Business and Economics University of Kentucky Lexington, KY United States; ^3^ University of Kentucky Rural Cancer Prevention Center College of Public Health University of Kentucky Lexington, KY United States

**Keywords:** social networks, social network analysis, personal networks, mobile surveys, survey development, survey implementation, low literacy, health disparities, rural health, Appalachia, cancer screening, diffusion of innovations

## Abstract

**Background:**

Personal social networks have a profound impact on our health, yet collecting personal network data for use in health communication, behavior change, or translation and dissemination interventions has proved challenging. Recent advances in social network data collection software have reduced the burden of network studies on researchers and respondents alike, yet little testing has occurred to discover whether these methods are: (1) acceptable to a variety of target populations, including those who may have limited experience with technology or limited literacy; and (2) practical in the field, specifically in areas that are geographically and technologically disconnected, such as rural Appalachian Kentucky.

**Objective:**

We explored the early-stage feasibility (Acceptability, Demand, Implementation, and Practicality) of using innovative, interactive, tablet-based network data collection and visualization software (OpenEddi) in field collection of personal network data in Appalachian Kentucky.

**Methods:**

A total of 168 rural Appalachian women who had previously participated in a study on the use of a self-collected vaginal swab (SCVS) for human papillomavirus testing were recruited by community-based nurse interviewers between September 2013 and August 2014. Participants completed egocentric network surveys via OpenEddi, which captured social and communication network influences on participation in, and recruitment to, the SCVS study. After study completion, we conducted a qualitative group interview with four nurse interviewers and two participants in the network study. Using this qualitative data, and quantitative data from the network study, we applied guidelines from Bowen et al to assess feasibility in four areas of early-stage development of OpenEddi: Acceptability, Demand, Implementation, and Practicality. Basic descriptive network statistics (size, edges, density) were analyzed using RStudio.

**Results:**

OpenEddi was perceived as fun, novel, and superior to other data collection methods or tools. Respondents enjoyed the social network survey component, and visualizing social networks produced thoughtful responses from participants about leveraging or changing network content and structure for specific health-promoting purposes. Areas for improved literacy and functionality of the tool were identified. However, technical issues led to substantial (50%) data loss, limiting the success of its implementation from a researcher’s perspective, and hindering practicality in the field.

**Conclusions:**

OpenEddi is a promising data collection tool for use in geographically isolated and socioeconomically disadvantaged populations. Future development will mitigate technical problems, improve usability and literacy, and test new methods of data collection. These changes will support goals for use of this tool in the delivery of network-based health communication and social support interventions to socioeconomically disadvantaged populations.

## Introduction

Social science research has established the powerful role that our personal social networks play in our lives [1]. The structure and content of our social networks provide (or restrict) opportunities for access to information and resources, social support, social capital, exposure to norms and behaviors, and other mechanisms that consequently impact our behavior, health outcomes, and success [2-5]. Our networks impact how long we live [6-8], how healthy we are [9-12], what health information and resources we have access to, and how we are able to use these to support our health [13,14]. However, in order to understand this structure and measure the impact of networks on the lives of individuals and communities, we need to properly collect and analyze network data. While data from online social networking sites can be obtained to analyze online network behavior, collecting data about a person’s *real* personal social network is an entirely different endeavor.

In ego network analysis (a subset of social network analysis) research focuses on an individual’s personal network, which consists of the immediate contacts (alters) connected to an individual subject (ego) [1,15]. For example, an ego network may consist of the people one individual relies on for social support, or seeks health advice from. This approach is different from a whole network or sociometric approach, which analyzes an entire bounded network (eg, the network of romantic relationships in a specific high school, or a network of organizations in a coalition advocating for health policy). In a sociometric study of a bounded network, each network member that is surveyed provides information about his or her self and his or her direct ties to other specific individuals who are members of the same bounded network. For example, a student is presented with a roster of all students in his or her grade, and is asked to indicate with whom he or she eats lunch with at least once a week, or seeks advice from for a specific topic. In an ego network study, the subject is expected to provide not only information about his or her self and his or her direct ties to alters, but also details about each of his or her alters and the relationships or ties between these alters.

To reach a wide and diverse sample of network members, some researchers ask broad *name generators* such as, “name 25 people you know” which may or may not be followed with a prompt for more specific relationships or context to consider during recall. After the names of the alters are generated, the subject is asked to provide descriptive information about each of the alters via *name interpreter* questions. This descriptive information is often sociodemographic (eg, gender, relative age, or education) or evaluative (eg, how much do you trust health information from this person, how frequently do you discuss personal matters with this person). These descriptive questions scale linearly with the number of alters. For each additional alter an ego provides, the ego must answer one additional response per question to describe the new alter. To ascertain some information about the structure of an ego network, the respondent may also be asked to describe the relationships between each *pair of alters* in their network. For example, if ego Bob names Mary and John as alters in his network, then Bob may be asked whether Mary and John talk to one another when Bob isn’t around. This allows researchers to understand which of the alters in the ego’s network are connected to one another as well as to the ego. However, the number of *alter-alter tie* questions scale with the square of the number of alters.

Network data collection often provides a tremendous burden on respondents who have larger networks, and as a result scientists may restrict the number of alters an ego can name (which may or may not decrease the richness and value of data collected, depending on the research question) or eschew network data collection entirely. In addition, this burden can discourage interviewers from allowing respondents to report higher numbers of alters, resulting in interviewer effects on network size [16,17]. Respondents may also be discouraged from naming alters, particularly in longitudinal studies in which a respondent may learn that naming fewer alters in subsequent interviews results in fewer questions, and minimizes the time needed to complete a survey.

Fortunately, in the past decade, advances in social network data collection and visualization software have reduced the burden of network data collection for researchers and respondents alike, particularly for ego or personal network studies [18-24]. The next step is to understand how well these advances work in the field, including whether they: (1) function as intended; (2) are able to accurately, reliably, and efficiently represent the personal networks of respondents; (3) are acceptable to a variety of target populations, including those who may have limited experience with technology or limited literacy; and (4) are practical in the field, specifically in areas that are geographically and technologically disconnected. While some investigation and testing of the functionality and data quality produced by these tools have been explored in the literature, the last two points—acceptability by target populations and practicality in the field—are of particular interest to researchers whose work is focused on reaching marginalized, socioeconomically disadvantaged populations, and connecting them to health services, support, and solutions.

We explored the feasibility of using interactive, tablet-based network data collection and visualization software (OpenEddi) in field collection of ego network data in Appalachian Kentucky, which is a geographically and technologically isolated population with population-levels of education and literacy well below the national average [22,25]. We followed the recommendations of Bowen et al [26] in assessing feasibility during an initial phase of intervention development. To do so, we want to answer the question, “ *Can it work?* ” The goal of this paper is to describe the acceptability, demand, implementation, and practicality of using this software in the context of a health communication study. Quantitative data was collected via implementation of the tool, and the qualitative experience of Appalachian research participants and interviewers was also examined. We aimed to demonstrate the benefits and challenges of using OpenEddi and a network visualization approach in this population and setting, thereby informing long-term goals of applying OpenEddi as a tool to facilitate the delivery of network-based health communication and social support interventions.

**Figure 1:**
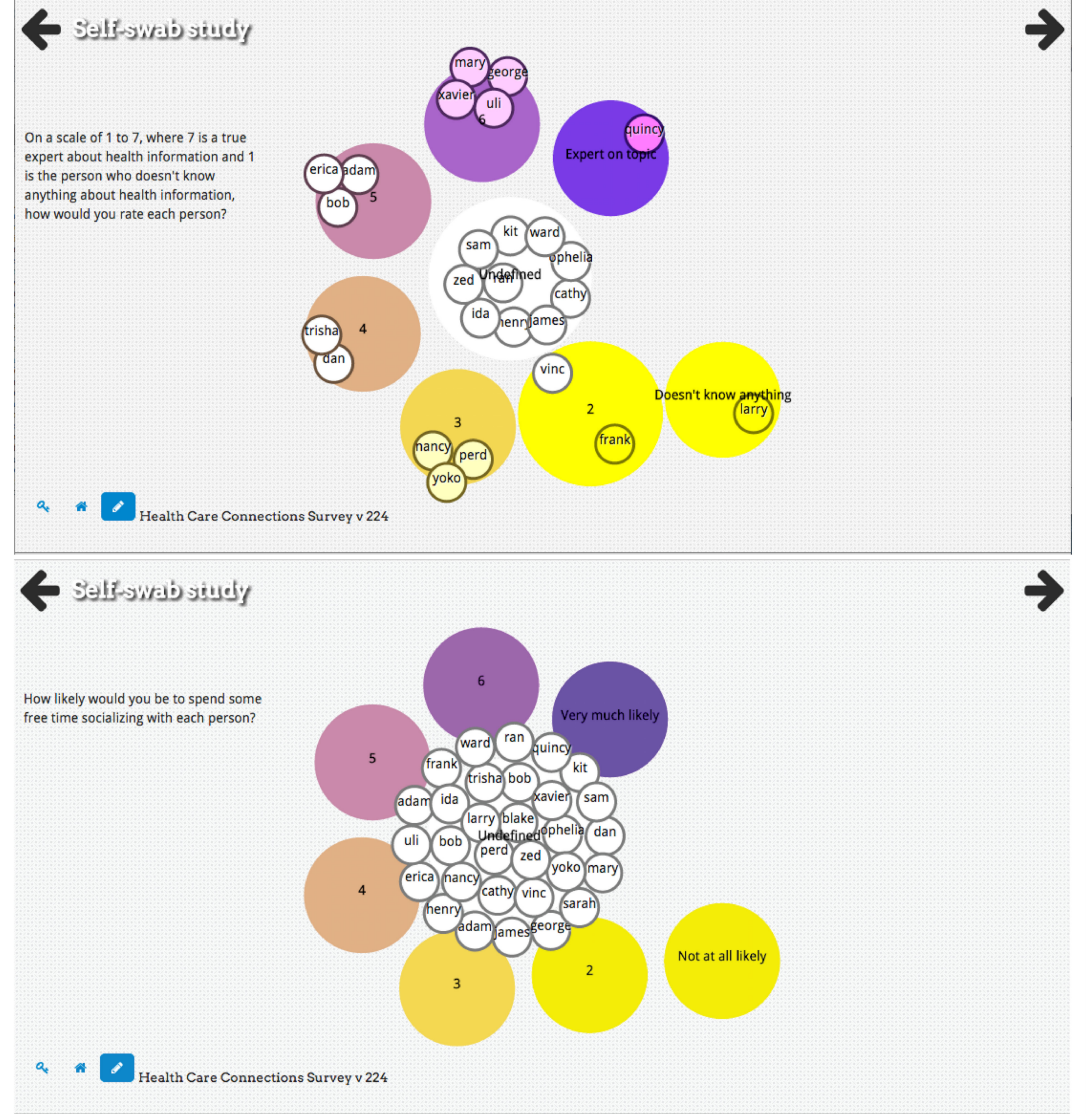
Examples of an alter characteristics bubble sort, which is used to assign alter attributes.

### Background

#### OpenEddi Data Collection Tool

**Figure 2:**
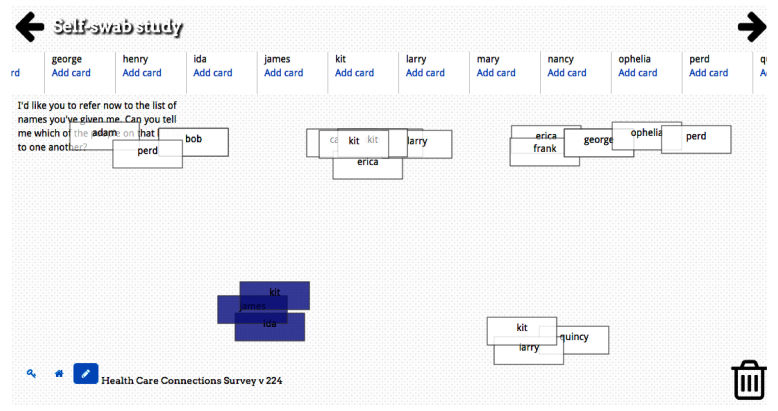
Example use of pile sort feature to elicit ties between alters.

OpenEddi is an adaptable, modular survey software platform designed for interactive, tablet- and mobile-ready field collection of network data, with or without an Internet connection. The platform was created to reduce the burden of network data collection on both the participant and the interviewer by using visuals to simplify the process of identifying and characterizing alters (Figure 1) and alter-alter ties (Figure 2), which may also improve reliability and validity of network data [27]. More detail on OpenEddi can be found in Multimedia Appendix 1. This paper reports the use of OpenEddi Version 0.2. We continue to develop and improve the software to best meet user needs. A video of Version 0.3, which is in development, can be viewed in Multimedia Appendix 2.

OpenEddi question types fit into five broad categories: (1) general survey questions or ego characteristics, (2) network identifiers (name generators, rosters, name interpreters), (3) alter characteristics, (4) relationship or tie characteristics, and (5) ties between alters. Alter attribute and relationship characteristic questions may be asked alter-by-alter (ask all questions about each alter before moving to the next alter) or question-by-question (ask a single question and respond for all alters before moving to the next question), or can alternate between both approaches. The program is ideally suited for the question-by-question format, which may produce more reliable and valid data [27].

**Figure 3:**
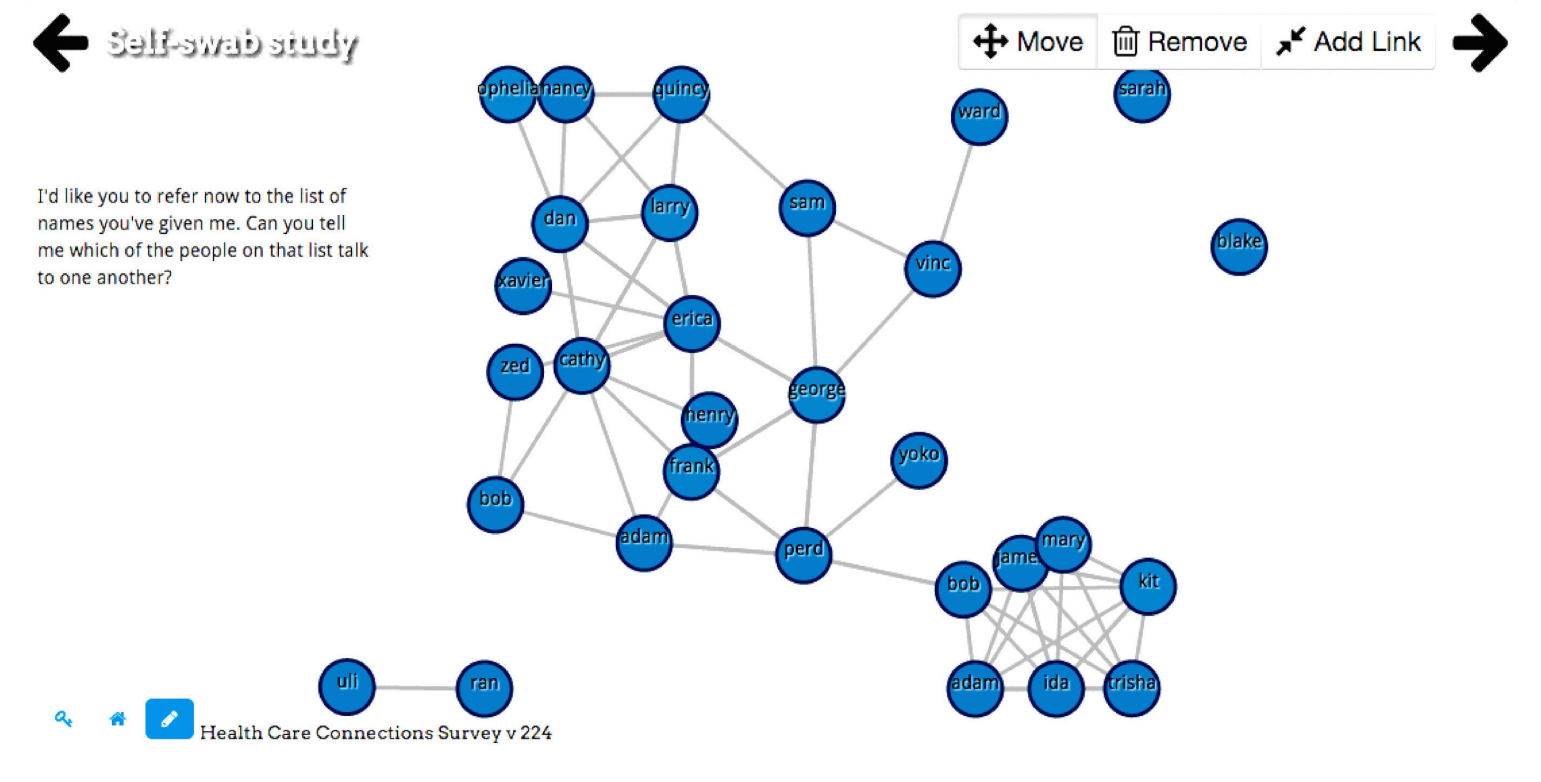
Example of nodelink diagram representation of participant network with ties between alters. Users may also use the nodelink diagram to add or delete these ties.

Creating a tool that is accessible to those who may have lower literacy, computer literacy, or education was an important goal in designing OpenEddi. User interface design for low literacy populations has focused on simplifying visual and audio approaches. OpenEddi employs a simple user interface design, including having *back* and *forward* buttons for navigation, as well as a *home* button rather than drop-down menus; this linear navigation can make digital tools more accessible for low-literate populations [28,29]. Radio buttons and large visual icons are recommended tools for use with nonliterate or low-literate populations [29], as are text-to-speech and audio input tools [30]. Colorful shapes, large buttons, questions that are answered by dragging bubbles into groups, and dynamic network graphical displays (Figure 3) are all examples of how OpenEddi uses playful design to gamify the data collection experience. Additional examples of OpenEddi’s user interface can be seen in Multimedia Appendix 3.

Other scalable, open-source mobile health data collection tools have been created with flexible platforms (eg, Survalytics [31], Beiwe [32]) for offline field collection of data using culturally appropriate means [33], and for using visual-based representations of standard survey question types in order to reach those with lower health or technological literacy (eg, Pit-A-Pat [34]). OpenEddi is the first platform to combine these three features with the ability to collect social network data (sociometric and personal) to create a powerful system for engaging populations who are often deemed “hard to reach” with traditional data collection means and methods.

#### Parent Study Population and Context

The easternmost third of Kentucky is considered part of the Appalachian mountain range, and contains some of the most unhealthy and poorest counties in the United States [35]. Kentucky has the highest rates of cancer mortality in the United States, and in the Appalachian Kentucky River Area Development District (KRADD), where our study was conducted, the cancer mortality rate is 41% higher than the national rate [36]. Associated with this unequal burden of cancer is the fact that Appalachian women are less likely to be screened for cancer within recommended guidelines, compared to non-Appalachians [37-42].

Education and health literacy are far below average in Appalachian Kentucky; 43% of adults in the KRADD have less than a high school diploma or equivalent, compared to 25.9% of Kentuckians and 19.6% of US adults [43], and this level of education is nationally correlated with having below-basic health literacy [44]. Some effort has been made to adapt health research and community engagement tools to address this communication barrier, yet there remains a need for easy-to-understand, enjoyable tools to reach populations with low education and literacy.

The mountainous terrain of Appalachian Kentucky, along with a history of economic depression, has resulted in a population that is geographically, technologically, and economically isolated, leaving Appalachian residents difficult to reach with roads, telephone, Internet, food, jobs, and health and social services. However, this same terrain shapes close-knit kinship and community ties, social cohesion, and a strong sense of native Appalachian heritage that result in powerful social support networks in Appalachian communities [45,46]. These networks, while strong in emotional support, are limited in access to formal social, health, and economic resources. Few studies have examined how network ties in Appalachian communities might be used to communicate information and norms about cancer screening and prevention behaviors, but those that do have uncovered network structures conducive to information diffusion by change agents and a willingness of Appalachian *key players* (or opinion leaders) to assume this role [38,47].

We conducted a pilot study to explore peer word-of-mouth communication networks in rural Appalachian women who interacted with an innovative screening method for high-risk strains of human papillomavirus (HPV) that cause cervical cancer, to better understand how to activate networks to disseminate innovative cancer screening and prevention information. After this study concluded, we conducted an informal qualitative group interview to obtain feedback on participant and interviewer experiences using OpenEddi. Quantitative data from the network study and its implementation, and qualitative data from the group interview, are examined in this paper to assess the feasibility of using OpenEddi to successfully collect social network data in a geographically isolated population of rural Appalachian women. Results will guide iterative development of OpenEddi as a method and intervention tool, moving toward further feasibility and efficacy trials.

## Methods

### Study Setting and Participants

The Rural Cancer Prevention Center (RCPC), a federally-funded Prevention Research Center, conducted a study in which rural Appalachian women used a self-collected vaginal swab (SCVS) for HPV testing [48]. This study will hereafter be referred to as the *SCVS study*. A total of 400 women between the ages of 30-65 years who resided in rural Appalachian Kentucky, were sexually active, reported no Papanicolaou test in the past three years, and reported never testing positive for HPV, participated in this study. Women were recruited through flyers in health departments, community outreach events, and other health care settings. Women in this study reported an average age of 40.2 years (standard deviation [SD] 9.3), most reported their ethnicity as white (94%), and most had a monthly income of less than US $1000 (59%).

We trained four nurse interviewers (all native to the KRADD and employed by the RCPC) to use OpenEddi to obtain information about participants’ egocentric social support and communication networks. This study will hereafter be referred to as the *network study*. Any woman who had participated in the SCVS study and agreed to be contacted for future studies was eligible for recruitment into the network study. Interviewers recruited individuals via telephone or in person. Women who agreed and consented to participate were interviewed using iPads at a location chosen by the participant between September 2013 and September 2014. Given very limited access to the Internet in rural KRADD, it was important that the interview and data collection could be conducted without an Internet connection. Interviewers administered the nonnetwork portion of the survey to participants verbally and recorded participants’ responses. For the network questions, the interviewer turned the tablet over to the participant and provided guidance as the participant navigated the survey interface herself. In some cases of very limited literacy or vision, the interviewer read most or all of the questions.

The final screen of the survey displayed the names the participant provided in response to a question regarding with whom she discussed the SCVS study. Participants were then provided cards with their study identification number and the interviewer’s contact information, with a request to have these identified network members call the interviewers to participate in the study, which resulted in the recruitment and enrollment of up to three of any participant’s network members. This *second wave* of individuals was recruited, and those who consented received one of two surveys: (1) if she also participated in the SCVS study, she received the identical survey administered to first wave participants; or (2) if she had not participated in the SCVS study, she received a similar network survey with differences in questions pertaining to the SCVS study (eg, eligibility for SCVS, why did she not participate if eligible) and the inclusion of sociodemographic information that we did not have access to via the SCVS study. Study participation concluded after the single interview session.

We conducted an informal qualitative group interview of all four nurse interviewers who had administered the network survey to respondents, and two women who participated in the network survey, to better understand the user experiences of OpenEddi in this setting. All of the women in attendance lived in the KRADD. The four nurse interviews comprised 100% of the interviewers in the network study. The two network study participants were recruited by the nurse interviewers to voluntarily participate in this qualitative group interview, to provide feedback on the software and their participation in the network study, and represent a small convenience sample of the total population of network study participants. We conducted this qualitative group interview in May 2015 at the Perry County Extension Office in Hazard, Kentucky. The authors served as moderators and took notes electronically to record responses. Open-ended prompts were used to elicit feedback from group members. There was no audio recording of the group. However, responses quoted in the results section are reported as accurately as possible. While the authors prepared an informal interview guide of topics to address in advance, the discussion evolved organically and was led primarily by the participants, with prompts from the authors. We did not develop formal coding strategies for the interview notes; the authors discussed the interview responses and synthesized themes together.

The University of Kentucky Institutional Review Board (IRB) approved both the parent SCVS study and the network study. While IRB approval was not obtained before conducting the informal qualitative group interview, permission was granted by the University of Kentucky IRB to use the resulting data in publications and presentations.

### Description of Network Survey

Nonnetwork questions (on topics such as health communication, health care access, social capital, political engagement, and innovative screening method for HPV) were asked first, followed by the ego network survey. A description of the ego network survey components, and how they are presented and used in OpenEddi, can be found in Multimedia Appendix 1.

### Feasibility Measures

We stated that the next step in developing innovative technologies for network data collection is to understand how well these advances work in the field. We used guidelines from Bowen et al [26] to assess this feasibility in four areas of early-stage development of OpenEddi: *Acceptability*, *Demand*, *Implementation*, and *Practicality*.

#### Acceptability

“To what extent is a new idea, program, process, or measure judged as suitable, satisfying, or attractive to program deliverers? To program recipients?” [26]. Outcomes of interest for acceptability of OpenEddi include: (1) are interviewers and participants satisfied with their experience using OpenEddi?; (2) is OpenEddi perceived as appropriate for use in a rural Appalachian population?; and (3) does the use of OpenEddi fit in the organizational setting of the interviewers?

#### Demand

“To what extent is a new idea, program, or measure likely to be used?” Outcomes of interest for demand of OpenEddi include: (1) would interviewers and participants prefer using OpenEddi to other survey administration methods?; and (2) do the interviewers perceive demand for using OpenEddi in implementing research?

#### Implementation

“To what extent can a new idea, program, process, or measure be successfully delivered to intended participants in some defined, but not fully controlled, context?” Outcomes of interest for implementation in this study include: (1) can OpenEddi be used to collect data for a network study in rural Appalachia?; (2) are there any specific resources needed to implement OpenEddi in this setting?; and (3) what factors affect OpenEddi’s implementation ease or difficulty?

#### Practicality

“To what extent can a new idea, program, process, or measure be carried out with intended participants using existing means, resources, and circumstances, and without outside intervention?” Outcomes of interest for practicality include: (1) was OpenEddi efficient in collecting data from participants?; (2) was the data collected using OpenEddi of good quality?; (3) were interviewers and participants able to use OpenEddi with ease?; and (4) did the use of using OpenEddi reveal any observed positive or negative effects on participants or interviewers?

### Analysis

Qualitative data are reported directly from the author’s notes and presented verbatim when possible. Descriptive statistics (eg, mean, SD, proportions) of ego data from the network study were analyzed using IBM SPSS Statistics Version 24 for Mac [49] and RStudio [50,51]. Network descriptives (size, number of edges, density, subnetwork proportions) were calculated using RStudio.

## Results

Results are organized by the four feasibility focus areas, using both quantitative data from the network study and qualitative data from the group interview. Responses related to acceptability and demand were often provided in tandem. Similarly, issues of implementation and practicality overlapped. Therefore, we combined these responses into two groups to report results. In addition to evaluating the practicality of OpenEddi, we included qualitative responses reflecting the practicality of the survey instrument administered in OpenEddi, and responses to the network visualization experience of OpenEddi.

### Acceptability and Demand.

Qualitative group interview respondents reported that they enjoyed taking and administering the survey:

We absolutely loved using the tablet.

Best survey I’ve ever done.

People loved doing this survey.

People were willing to do it and it was easy because it’s just moving circles around.

In addition, respondents reported that the survey was engaging, easy to use, and that they liked it better than other survey software they had previously used:

Our survey was engaging and they had to think about questions and not the same thing over and over.

It’s more engaging to think about friends and family than yourself. You have to think about the people you know.

Other software is really challenging.

School software is a lot more boring than this. It’s all fill in the blank and strongly agree.

Respondents reported that the bubble sorting of alters was fun:

It was fun like a video game!

It was a lot more interesting and not the normal boring select one, and after three you want it to be over.

However, the questions became repetitive, and interviewers suggested mixing up the structure and interchanging the bubble sort questions with standard multiple-choice questions:

Oh gosh another circle?

People got tired of circles so if you could change up the structure that might be better. Mix it up.

Interviewers stated that they wished they could use OpenEddi for all of their surveys. One interviewer expressed an understanding of how the software and network visualizations could be used in another intervention study she was involved in:

If we make people aware and spread information, it’s beneficial to know who knows each other. For the FIT [fecal immunochemical testing] kits, if we had access to the survey networks we’d have access to these other people.

There were usability issues in administering the survey to some participants with low or no literacy, including difficulty reading a smaller font size:

People who had never used an iPad either thought it was fun or they had a hard time. Even after increasing font size they couldn’t read it. Younger people had an easier time than older.

I had to read a lot of them.

When qualitative group interview respondents were asked, “what would you change?” the responses were mostly aimed at: improving the visibility and literacy of the survey, including being able to choose a font size, color options, and shapes; having colors assigned to alter attributes (so that the bubble representing an alter would be a color that reflected an attribute assigned to that alter); and “anything to expedite the sorting!” One recommendation was to group alters by type of relationship before entering the pile sort exercise to make alter-alter tie connections easier, or to ask subsequent questions on a similar subgroup.

### Implementation and Practicality.

Qualitative group interview responses related to implementation and practicality are reported first, followed by results from quantitative data collection.

#### Qualitative Group Interview Responses

The fact that an Internet connection was not needed to administer the survey was repeatedly brought up as a great benefit to using the survey software, but there were sometimes issues with syncing the data when an Internet connection was established. One respondent stated, “[Not needing Wi-Fi] not just made it easy, it made it *possible!* But there were some difficulties with syncing. *”* There were events of surveys not saving data after an entire survey was completed (“People had to re-do it. They were not happy *at all*.”) and occasions when the survey would, “…kick you out and when you’d get back in, you could put the page number in the URL. I’d pay attention to what page it was on so in case something happened they could go back in and restart where they left off.”

In relation to usability of specific features of OpenEddi, respondents reported that the pile sort was somewhat helpful, but hard to understand, and sometimes the software didn’t work properly during this portion of the survey.

The rectangles [cards with alter names] gave you a head start…

This was confusing, linking the squares on top of each other. Sometimes they wouldn’t let you touch them.

The interviewers made several suggestions for improving the pile sort:

Use the questions leading up to the pile sort to group people together so that you aren’t starting from the beginning sorting people again.

Make something where all the people in this column talk to all the people in that column. “Select all” for one column means everyone talks to one another.

Similarly, issues arose with the graphic user interface of the nodelink diagram. One respondent mentioned the lack of distance in the graph, making the network difficult to visualize: “It was hard to see how it looked, like we’re all together, because it pulled all the circles – so if it stayed further apart it would have been easier to see.”

However, most responses were reflections on what the graph represented rather than the utility of the software. Respondents were surprised at how densely connected their networks were, or at the size of their network:

Mine was a big group of people who were all connected and it was really close knit.

Surprise. I’m surprised I talk to all of these people.

Other respondents found that the visualization made them aware of what their network looked like, who was in it, and how it impacted her life:

It was a struggle to come up with a lot of people…the people I communicate with is really limited…

My group is really small and I really need to branch out… if I was doing something for health or wellness I could see who I talk to for certain things...

One interviewer mentioned that she had a participant who, after noticing that her network had a main core group and a couple people outside of it, realized that she was connecting two groups within her family that didn’t communicate with one another. She stated, “I don’t know why these people don’t talk to other people because they talk to me.” She was surprised by that. It was her family, and two people in the family who didn’t talk to the rest and, “if it weren’t for me they would never talk to each other,” and she didn’t realize it until she saw the diagram. “Maybe I should do something to make them talk to each other instead of relying on me to link them together.”

One interviewer recommended using visualization, “to show the support they give you or show you who you talk to about certain things. I talk to these people about business things, and these about health.” Other responses reflected the practicality of the survey that was administered via OpenEddi in this study. In response to the name generator question, “Name 25 people you know,” respondents generally felt that 25 was, “a lot of people to come up with,” it took a long time to name them all, and they were confused about who these 25 people should be. Interviewers reported having to ask participants to think of coworkers and other friends as people in their network because, “they only thought of kin.” Respondents reported that after naming 25 people, participants really didn’t want to add anyone else to the network.

Without prompting, the respondents reported their surprise at how few participants in the survey know the age and education level of those close to them: “These are their best friends and family and I don’t know how old they are or level of education? How good friends we are and we don’t know these little things?”

In addition, respondents reported participant reactions to multiple questions about trusting network members and others in the community.

People were really tore up about the who do you trust questions. They had close family members and friends and they would say that they only trust one person.

Who trusts you? That would be hard to answer. They’d have an emotional break down and say, ‘I DON’T KNOW!’

One participant suggested that you could use your network graph to help select a person to trust who was less connected to the rest of their network, and would therefore be more likely to keep a secret: “You can look at your network! And pick that person way out there to tell something to!”

### Quantitative Network Study Data

Our goal in the network study was to collect data from 160 women–40 initial participants (seeds) and up to 3 alters from each seed’s network–and investigate whether referrals into the SCVS study flowed through women’s health information networks or resource provision networks. We interviewed a total of 168 women, 71 (42.3%) of whom were initial seeds from the SCVS study, and 97 (57.7%) of whom were network members of those initial seeds. The nurse interviewers found that recruiting the network members was more challenging than the initial seeds, although the proportion of initial seeds to alters recruited varied by interviewer.

There were technical issues with syncing the data when a Wi-Fi connection was established, and in saving the data locally. This first instance of OpenEddi used in the field (Version 0.2) was built on third party libraries for Application Program Interfaces (APIs) and database management to hasten the implementation of the software in the field. When the bugs inevitably emerged that affected the storage and retrieval of data, it became very difficult to locate and resolve the issues, and a portion of the collected data was lost.

In our final dataset, we have data from 84 egos (50.0% of 168 interviews), with data on 1750 alters, and 8698 ties between alters. However, due to the nature of the data loss, we can only link 53 egos (39% of total interviews) to their alters and alter ties in the dataset, giving us only 53 complete networks. Furthermore, we lost the identification data that we needed to link the initial seeds to the participants, so we were unable to distinguish which of the 53 egos were initial seeds and which were network members recruited from those seeds. Finally, of those 53 egos for whom we have complete network data, only 33 have complete ego data, meaning that for 20 of the 53 egos we have data on their alters and the ties between alters, but their individual-level survey data is among the lost data. This problem greatly limited the analyses we could conduct and the conclusions that can be drawn from the quantitative data.

We can use data from 84 egos to explore nonnetwork inquiries. Of the 84 egos, 50 (60%) had participated in the SCVS study. We had planned on linking our data from women who had participated in the SCVS study to the SCVS study data, so we only have sociodemographic information from the 34 women (40%) who *did not* participate in the SCVS study (shown in Table 1); most were non-Hispanic (99%), white (100%), had less than US $1000 per month in income (60%), and had children (73%). These results are representative of the population of the KRADD, which is 97.2% white and 0.6% Hispanic, and the median annual household income is US $28,022.

**Table 1:** Characteristics of network study participants, n=84 unless otherwise noted.

Variable		n (%)
Use the Internet or email at all (n=79)		67 (80)
**Frequency of Internet or email use (n=67)**			
	Daily use	41 (61)
	Weekly use	23 (34)
	Less often	3 (4)
**Devices used to access the Internet (n=67)**			
	Desktop computer	32 (48)
	Laptop computer	44 (66)
	Mobile phone or smartphone	56 (84)
	Tablet computer	30 (45)
Participated in SCVS study		50 (60)
*Demographic characteristics of network study participants who did not participate in the SCVS study, n=34*	
Age, mean (SD)		36.9 (14.1)
Race white (n=34)		34 (100)
Hispanic ethnicity (n=30)		1 (1)
**Monthly income (n=30)**			
	Less than US $1000	18 (60)
	US $1000 to $2000	9 (30)
	More than US $2000	3 (1)
Have children (n=30)		22 (73)
Have children under age 18 living in home (n=29)		16 (55)
*Structural characteristics of networks, n=53*	
Size, mean (SD)		12.6 (8.8)
Density, mean (SD)		0.67 (0.32)
Proportion of networks with density = 1		23 (43)

We can use data from the 53 complete networks to look at structural network characteristics (ie, size, number of edges, and density) and data on the alters and alter ties that exist in those 53 networks (see Table 1). Network size ranged from 2 to 27, with a mean of 12.6 alters. Network density, or the proportion of ties between alters present out of all possible ties between alters, ranged from 0.13 to 1. A network density of 1 results when every alter is connected to every other alter, which was observed in 43% of the networks in this study. In this study, ties between alters were defined loosely by the question, “Can you tell me which of the people on that list talk to one another?“

**Figure 4:**
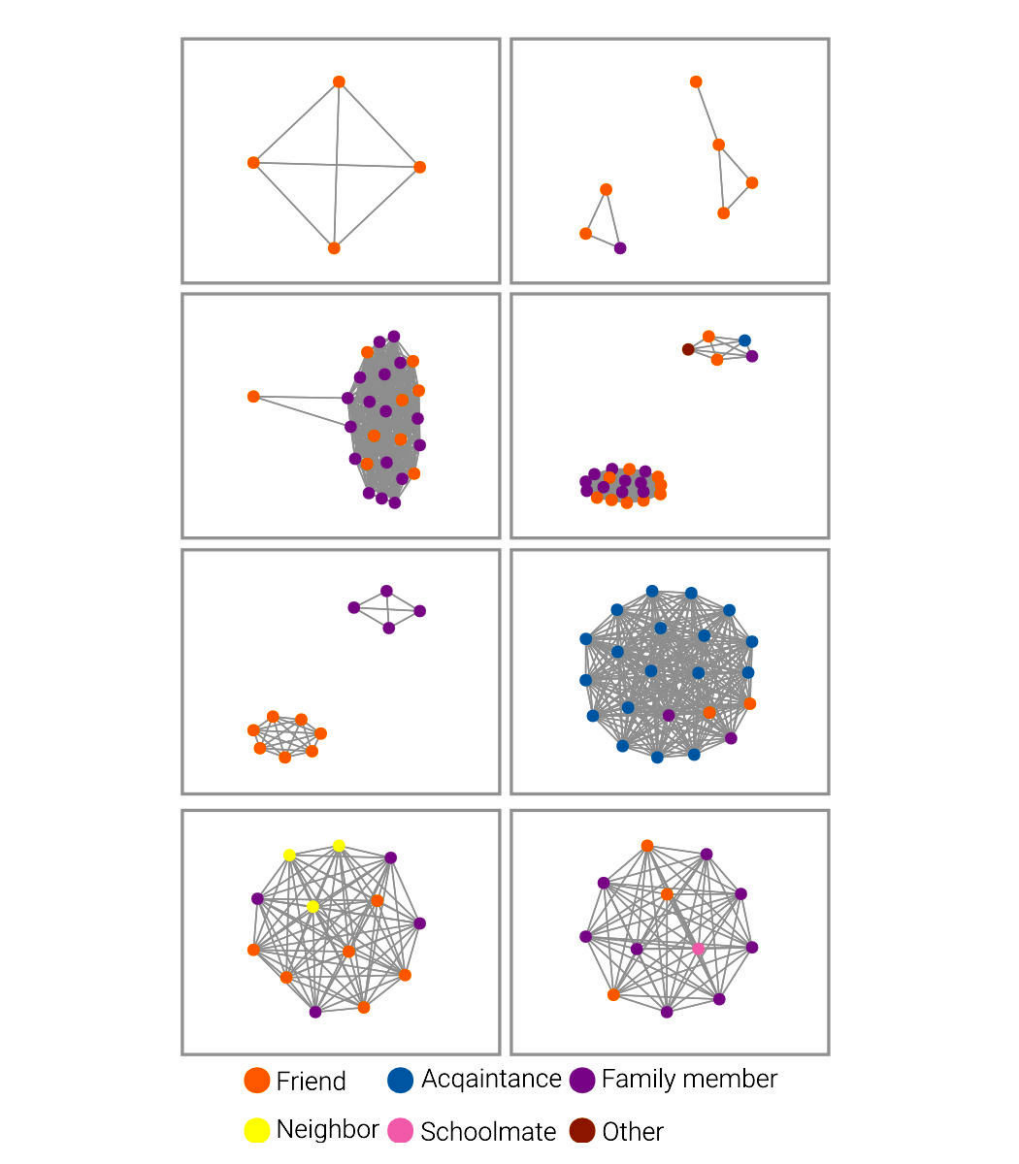
A random sample of 8 participant networks with node color representing type of relationship reported for each alter.

Figure 4 shows a sample of 8 participant networks, chosen at random. The circles are nodes, which represent network members. The grey lines are ties between network members. The ego is not included in the network diagram. In this figure, colors reflect the category of relationship the ego indicated for each alter. Most of these networks (5/8) show a single connected component, but three of them have two components, demonstrating that the ego has two groups of people in their network who do not interact with each other.

**Figure 5:**
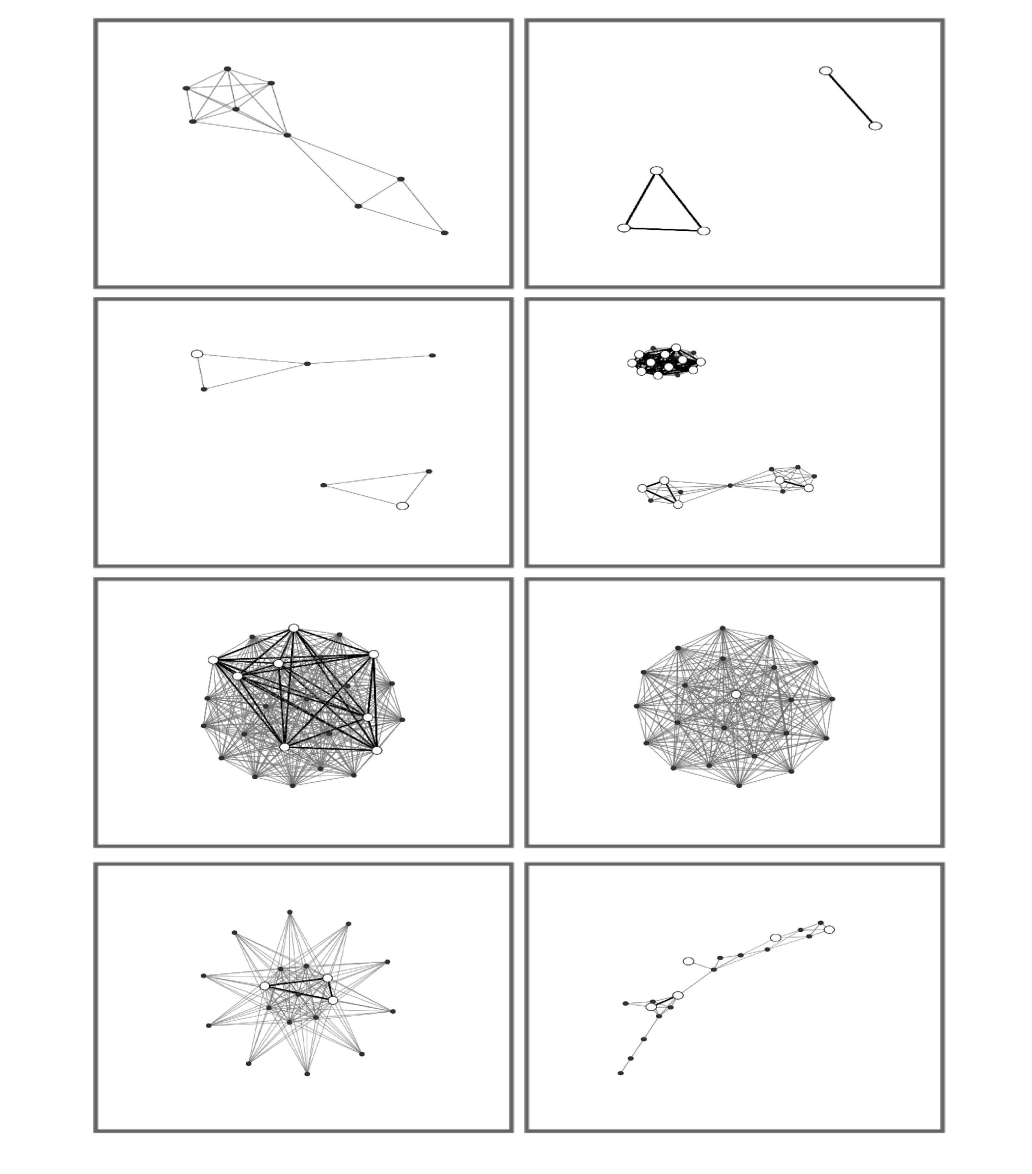
A sample of 8 participant networks with white nodes indicating alters with whom the ego talks about certain topics.

In the survey, we asked women with whom in their network they talk about health topics such as HPV, the self-collected vaginal swab, and cancer, and also with whom they talk about where to get things they need for the best price (a proxy for market mavenism). Figure 5 displays a sample of 8 participant networks with the white nodes representing alters with whom the ego reported talking with about any of the above topics. Darker lines indicate a tie between two of these alters. As demonstrated, some egos didn’t talk to any of their alters about these topics, and some talked to all of their alters. The proportion of the network with whom they communicated about these topics varied across egos. Further analyses will explore these subnetworks in-depth.

## Discussion

The purpose of this paper is to report on the early-stage feasibility of the OpenEddi data collection tool in a rural Appalachian population. In a qualitative group interview of a limited number of users, we found that OpenEddi was well received, useful, didn’t feel burdensome, and was fun to use. However, technical issues hindered the implementation of OpenEddi, and areas were identified for improved practicality of the tool for lower literacy users.

Due to the amount and type of data lost, we were unable to investigate our initial hypothesis about the type of network women accessed to recruit others into the SCVS study. It has yet to be determined what conclusions, if any, we may draw from the network study’s remaining data. We plan to analyze and present detailed survey data from the 84 egos in a separate paper, as well as the complete network and survey data of 33 egos. The descriptive data of the personal networks of rural Appalachian women, and the rich data we collected from egos on topics such as health communication sources and networks, health care access, social capital, political engagement, and use of an innovative screening method for HPV, will still fill an existing gap in the literature on networks of rural Appalachian women.

To address the technical issues that led to data loss, we have completely rebuilt the OpenEddi data collection platform in a more reliable way. The software has been rebuilt from the ground up using a more appropriate set of tools for storing and retrieving data: NodeJS, a custom API built using Express 4; and a PostgreSQL database, which made wide use of the JavaScript Object Notation data type. Not only is this new version (Version 0.3) easier to debug, it also better suits the adaptable vision of the software.

Most of the time, we were successfully able to collect data in people’s homes and places of employment (even at a Dairy Queen), without an Internet connection, in a rural and geographically isolated area of the United States. The nurse interviewers felt that we would simply have been unable to conduct the study if an Internet connection had been required. Collecting data at a designated office, or in the homes of people who had a computer with an Internet connection, would have severely limited the diversity of socioeconomic status, technology use, and geographic location of our study sample. Having a mobile data collection tool allowed the interviewers to survey participants wherever they were, which not only broadened the reach and representativeness of the sample but also sent a message to underrepresented and understudied participants that we, as researchers, value their time and their perspectives.

We did not formally assess respondent and interviewer fatigue or completion time in this study. However, the nurse interviewers using OpenEddi greatly appreciated that they did not have to enter the data from paper-and-pen surveys, and even when the technology failed, they felt it was less burdensome than other surveys they had administered. Of note, none of the interviewers had ever administered a network survey, yet they still preferred the network survey to any standard survey they had previously administered. Respondents reported that participants in the network study were excited to use the tool, regardless of familiarity with technology, and found it to be fun, like a game. There was a learning curve for some participants unfamiliar with touch-screen technology, but more barriers were raised in response to basic literacy needs such as changeable font size, shape and color of nodes, and text-to-speech options.

Our research team has identified these issues of literacy as a primary area of focus for the development of OpenEddi. Respondents believed that a text-to-speech option would be beneficial, but insisted that it be an option in the software for each question, not that the entire survey be delivered only via audio. We recommend interviewer-guided data collection in populations with potentially low literacy to assist participants with understanding and navigating software, to provide a more personal experience, and to be able to identify struggles with literacy and compassionately help participants engage in the research.

Respondents in our study found the visual-based bubble sorting procedure and nodelink diagrams to be fun and easy to understand. The pile sort, in contrast, was difficult for participants to use without explicit guidance. The pile sort procedure may not have matched the way participants cognitively stored relationships between network members, or the layout of the task on the screen may have been too complex. We have designed additional methods for collecting alter-alter tie data, and plan to design and test additional approaches to structuring survey questions among populations with varying levels of literacy. By using nonlinguistic graphics and creating visual representations of common response scales we may better reach low-literacy users while enhancing the experience for all users.

The participants in this qualitative group interview had excellent suggestions for reducing the burden on survey users by expediting sorting of (or characterizing) alters and identifying alter-alter ties. We now offer four different methods of eliciting alter-alter ties in OpenEddi: (1) the pile sort; (2) the nodelink diagram; (3) the box pop, which is a unique variation on the traditional pair list (listing every pair of alters and asking if they have a tie); and (4) an alter grouping method in which alters can be assigned to groups of people who are all linked based on some attribute (eg, family, coworkers, attended an event). Any of these approaches can be used as a method on its own to identify alter-alter ties. If visualizing the network is a goal of the data collection process, any of these approaches may be used to first elicit alter-alter ties, then the ties can be visualized as a nodelink diagram. Alternatively, the nodelink diagram can be used alone to both elicit and visualize the alter-alter ties.

In a randomized trial comparing the first three of these OpenEddi methods (the alter grouping wasn’t yet fully developed) with two established computer-based alter-alter tie methods (pair list and matrix) and two paper-and-pen methods (paper matrix and paper nodelink), we found that these methods yielded significantly different results on some measures (unpublished data). However, the OpenEddi nodelink diagram stood out as the favorite, scoring highest on all user satisfaction measures. Finally, we have changed the springiness of the force-directed graph in the nodelink diagram, which may reduce crowdedness and improve participants’ ability to visualize and comprehend their networks.

Perhaps the greatest discovery in this informal feedback session was the impact of visualizing network structures on the participant. Participants reported understanding their own networks better and seeing how links (or absence of links) impacted their lives. The respondent who was surprised at how small her network was and that she didn’t trust many people felt that this information allowed her to see where she could make changes to improve her social support network. Another respondent saw how she could leverage her network structure for a specific opportunity. She identified an isolate in her network (“that person way out there”) as the person to talk to about an issue she wanted kept secret, because she saw that node wasn’t connected to anyone else in her network. Upon the realization that she was the only person linking two disconnected factions of her family, one participant felt that she shouldn’t be the only “go-between” and that she needed to stop playing that stressful role.

Although we did not design the study to make use of the responses that the network graph might produce in participants, this approach presents opportunities to empower women from a resource-poor community to make changes in their own social networks. Using personal network visualization and feedback, we can tailor interventions to individuals by identifying areas of need and connecting them to new resources and information, tools for building social support, or other strategies for maximizing the positive support and resources available in their network [11,52-55].

There are exciting projects ongoing in the field using this approach, such as using motivational interviewing (MI) with network visualization to reduce risk behaviors among persons transitioning from homelessness to stable housing [56]. In this intervention, for instance, an individual can see clearly through network visualization who in their network uses or encourages use of substances, and the interventionist can use MI to help the individual work through strategies to disconnect from those network members, or create stronger ties between network members who positively support the individual. In addition, Kennedy et al found that network visualizations can be used in populations with less than high school levels of education [53,57]. Dhand et al [14] are using the personal network characteristics of neurological patients to understand how network ties impact stroke outcomes, and propose additional research building clinical, evidence-based network interventions for neurological patients. We are developing a clinical network intervention for cancer patients, survivors, and caregivers to improve social support and resource provision over treatment and survivorship.

Based on the qualitative group interview data, we don’t know whether the reflections of the respondents on their network structures led to any network changes, but they do indicate that there is potential for this type of intervention. In our study, if the interviewer had been trained in MI, she could have helped the participant who no longer wanted to serve as the only link between family factions develop specific strategies to do so, by building self-efficacy to make that network change. On a population level, network visualization can potentially increase the social capital of a community by enabling individuals to see, understand, and leverage resources and support in their networks that they may otherwise be unaware of. Interventions using community-based network models (like lay health workers and change agents) have great potential for success in Appalachia [42]. For those researchers interested in reaching marginalized populations with resources and skills to improve their opportunities to be healthy, we hope to couple these community-based interventions with more tailored network feedback to individuals by arming community health workers with interactive network visualization software and training in MI. Future research should investigate this possibility.

### Limitations

This is the first study evaluating the use of a mobile network data collection and visualization tool in a rural Appalachian population, but there are several limitations. The qualitative study reported in this paper is limited by a very small sample size of only six individuals. Although this group captures 100% of the interviewers involved in the network study, the two network study participants only represent 1% of our total network study sample, including those whose data were lost. We appreciated that the interviewers could relay the experiences and comments of the participants they interacted with in administering the survey. In addition, the two women who did participate were known acquaintances of the interviewers, who agreed to volunteer their time, which represents a convenience sample. We used this approach because our original intent was to gain informal feedback to inform our continued development of the software, not to share the results. This issue limits the generalizability of our findings, and drives us to conduct additional research to further investigate the feasibility of OpenEddi.

In addition, the qualitative group interview took place nearly eight months after completion of data collection in the network study. The participants in our group interview were relying on their memory of the software and survey experience. This issue limits our ability to evaluate some of the nuances of using the software, but participants remembered their individual experiences with the software even after an extended period of time; some with very compelling stories of the impact of network visualization on their perception of their networks. Our next assessments of OpenEddi feasibility in the field will include a formal structure for recruitment, implementation, and qualitative data analysis. In addition, we are incorporating iterative usability testing and evaluation of the software as we continue to develop OpenEddi.

### Conclusion

This paper presents observations on the feasibility of using a touch-screen, tablet-based network data collection tool in a predominantly low-income, low-education population of rural Appalachian women. Qualitative feedback and implementation data have shown us that our tool, OpenEddi, has the potential to be successfully used in this population, yet there are improvements that must be made to further enhance the network science experiences of this group. Our next steps are to incorporate the feedback provided into the survey tool. We have rebuilt the OpenEddi software platform and are currently creating and testing new methods of question display. We have developed and tested several new methods of obtaining ties between alters (unpublished data). As we develop and test these methods, we will evaluate the quality, efficiency, and acceptability of these new mechanisms in comparison to existing network data collection methods.

Specifically, we plan to directly compare multiple data collection methods by implementing an identical full survey via OpenEddi, at least one established computer-based network data collection method, and at least one established paper-based method. We hypothesize that among higher-literacy users, data quality will be similar across methods, but that satisfaction and efficiency will be highest among the OpenEddi users. Among low-literacy users, we hypothesize that data quality, satisfaction, and efficiency will be highest among the OpenEddi users. By using playful design, we hope that a more enjoyable survey experience will lead to participants committing to survey completion and engaging in the survey process again for longitudinal studies, resulting in more accurate and reliable network data and attitudes towards engaging in network research.

The ability for existing scales and measures to be adapted to a visual representation while retaining validity and reliability is promising (eg, iPadVAS [58]). We will continue to develop and test visual-based question types and modules created specifically for acceptability and use in low literacy populations. A tool like OpenEddi can enhance community-based research in areas such as rural Appalachia by reaching underserved populations with effective information and connecting them to health and social services. Finally, we will continue to develop specific applications of the OpenEddi data collection and visualization tool in community-based and clinical settings for evaluating and intervening with social support and communication networks.

## Appendix

Multimedia Appendix 1 Description of OpenEddi design and network survey, with examples and screenshots of the OpenEddi survey.

Multimedia Appendix 2 Video demonstration of sample network data collection features of OpenEddi Version 0.3 (rebuilt version).

Multimedia Appendix 3 Additional features or question types of OpenEddi Version 0.2 that are currently being incorporated into Version 0.3: a) The bullseye feature represents closeness of alters to the ego; b) and c) demonstrate additional ability to answer questions by ego or by using a predetermined roster of names; d) network members or alters named in response to a specific question (eg, "With whom did you talk about the HPV self-swab study?") are brought up for recruitment into the study, to ease the ability to contact network members for snowball sampling or respondent-driven sampling.

## Abbreviations

API: Application Program Interface
HPV: human papillomavirus
IRB: Institutional Review Board
KRADD: Kentucky River Area Development District
MI: motivational interviewing
RCPC: Rural Cancer Prevention Center
SCVS: self-collected vaginal swab
SD: standard deviation
