# Supplementary material for: An Interactive, Mobile-Based Tool for Personal Social Network Data Collection and Visualization Among a Geographically Isolated and Socioeconomically Disadvantaged Population: Early-Stage Feasibility Study With Qualitative User Feedback
Source: JMIR Res Protoc. 2017 Jun 22;6(6):e124. doi: 10.2196/resprot.6927 (PMC5500782; doi:10.2196/resprot.6927)

Multimedia Appendix 1: Description of OpenEddi design and network survey, with examples and screenshots of OpenEddi survey

Designed with network data in mind, OpenEddi uses tools designed specifically to make filling matrices easier. The format allows for collecting data for either large or small sociometric studies (e.g., starting with a roster) or egocentric network studies (e.g., starting with a name generator), and can directly output data to network data formats such as UCINET or Gephi or feed directly into R [25-27]. There is also a specific “read.openeddi” import function within the egonetR package for egocentric network analysis in R [28]. The single-page web app design allows the tool to move easily and quickly at the respondent’s pace, and allows for data collection anywhere, as all survey resources cache to the tablet (or other mobile device) and sync when the device is connected to Wi-Fi. The clean, responsive presentation adapts to smartphones, tablets, or desktop computers, and the dynamic visualization allows one to build questions in brand new ways. As open-source software, a goal of OpenEddi is to encourage a community of development and innovation in data collection, resulting in a marketplace of data collection ideas and plug-ins.

For the network study described in this paper, the non-network questions (on topics such as health communication, health care access, social capital, political engagement, innovative screening method for HPV) were asked first, followed by the ego network survey.

*Name generator, name picker, network additions***.** We used a broad name generator (“Name 25 people you know.”), to prompt the participant (or ego) to enter a list of names (alters). Then, a series of “name picker” (name interpreter) questions allow the participant to more finely characterize communication relationships with alters, while also allowing for additional alters to be added. An example name picker question is: “Of the people you’ve named, whom, if anyone, do you seek out for health information you can trust?” The participant can select the relevant alters from a list, and add new names to the network if needed (see Figure 1, below).

Multimedia Appendix 1, Figure 1. Name picker (a) and name addition (b) examples, in which respondents can select names or add names to the network.

a)

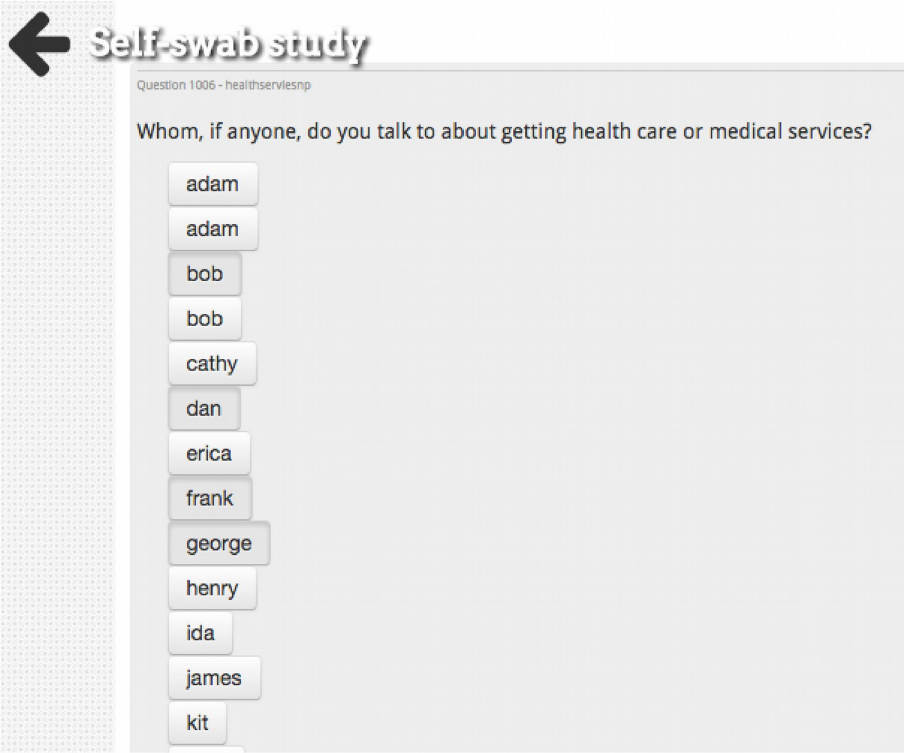

b)

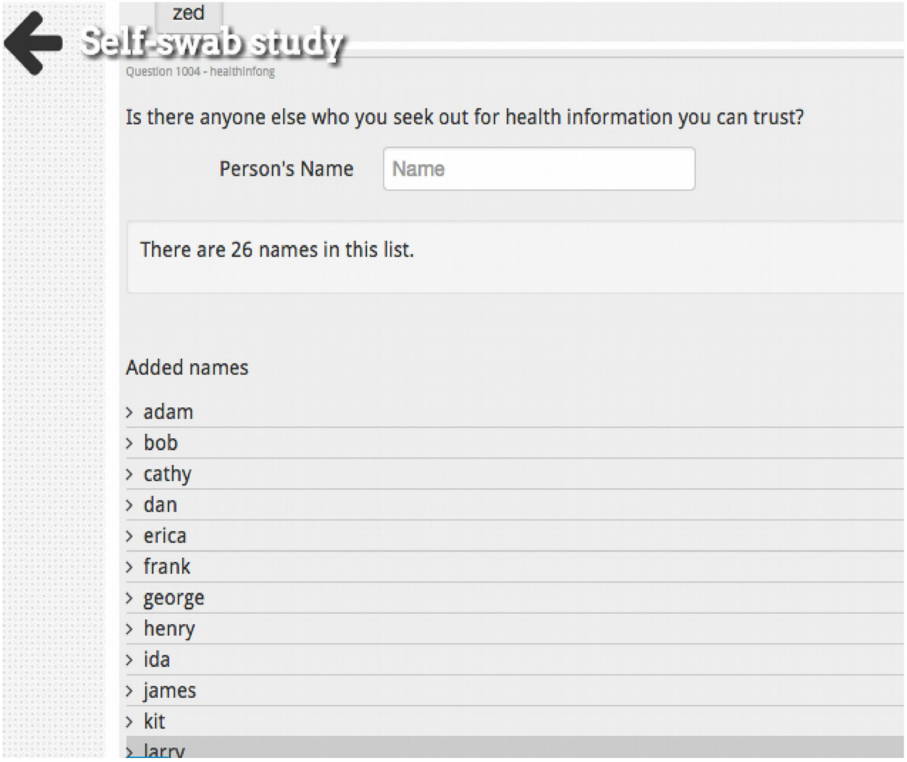

Alter characteristics and ego-alter relationships (ties). In order to understand more about the ego's network, we ask the ego to describe alters and the relationships, or ties, they have with their alters. In this survey, questions characterizing alters and ties involved sorting bubbles into multiple circles that represent response options. Participants respond by using their finger to move a bubble with the name of each alter into the appropriate response category. Examples of this are, "How do you know each person?" with circles for “family,” “friend,” “coworker,” etc., or "How likely would you be to spend some free time socializing with each person?" with 7 circles representing response options from “not at all likely” to “very much likely,” with scale ends labeled. See examples in Figure 2. A traditional survey response format and alter by alter method was used when the text of response options was too long, or did not fall into neat categories.

Multimedia Appendix 1, Figure 2. Examples of alter characteristics bubble sort, to assign alter attributes.

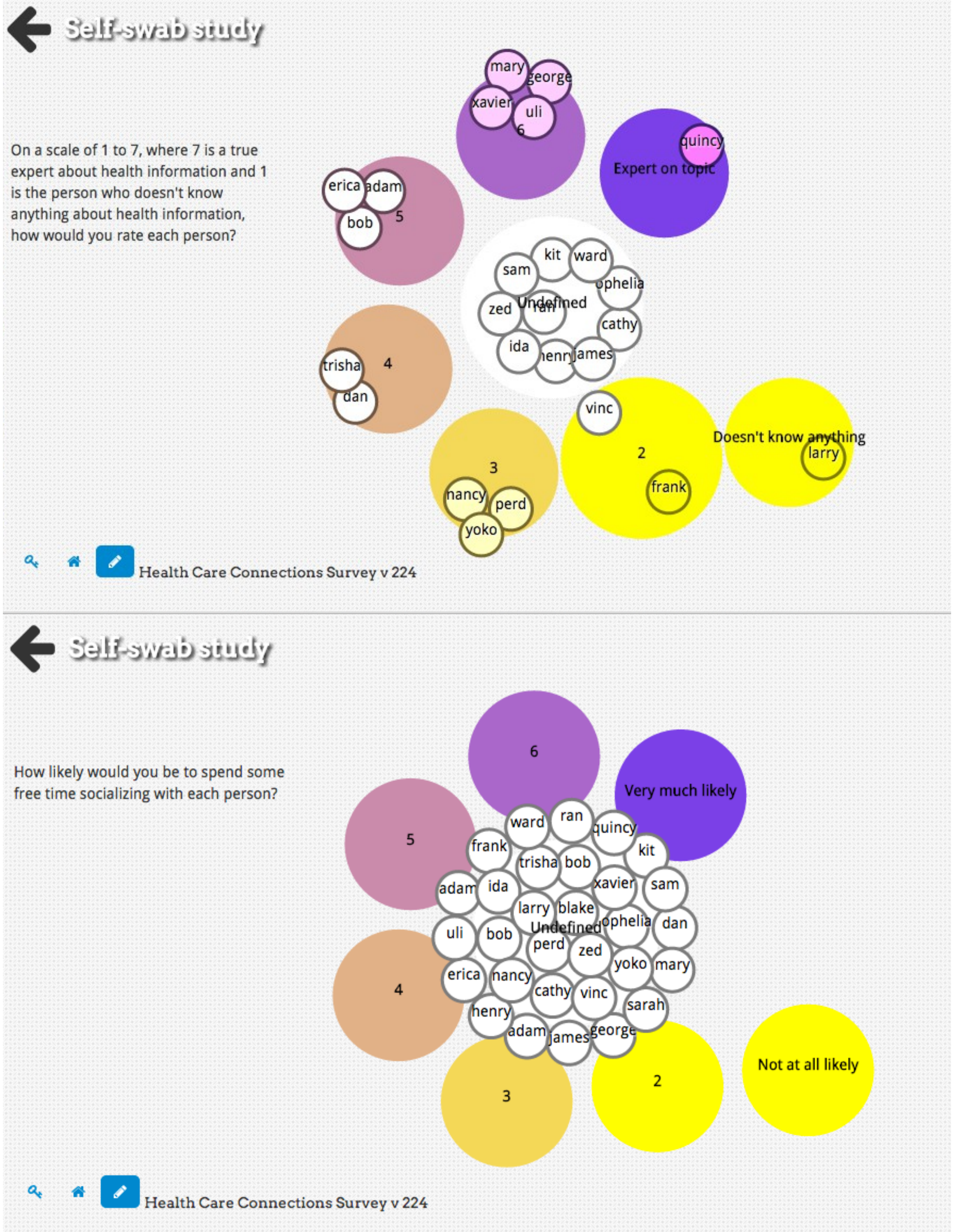

Ties between alters -- Pile sort. Finally, each participant is asked to use a pile sort technique to understand which alters interact. As seen in Multimedia Appendix 1, Figure 3, each alter is represented by a card at the top of the screen. In response to a prompt ending, "…Can you tell me which of the people on that list talk to one another?" the participant adds name cards to the screen and can create groups or piles of alters indicating connections between those alters. The same name can be used as many times as needed, and many piles can be created.

Nodelink diagram. A visual representation of the participant's social network is created using the ties between alters elicited in the pile sort exercise, with a force-directed layout (Multimedia Appendix 1, Figure 4). This nodelink diagram displays all connections made in the pile sort and allows for the participant to make real-time changes to their network graph by adding or deleting ties. It is also responsive to interaction: The participant can rearrange the network into different displays by simply dragging their finger on the touch-screen.

Multimedia Appendix 1, Figure 3. Pile sort example to elicit alter-alter ties.

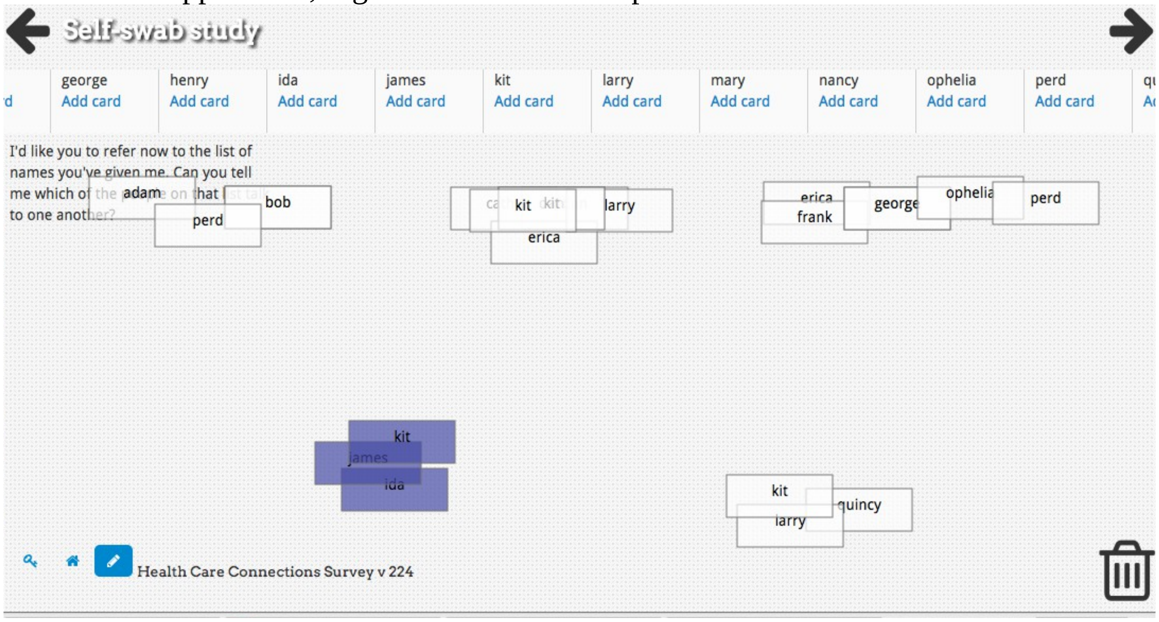

Multimedia Appendix 1, Figure 4. Nodelink diagram representation of participant network

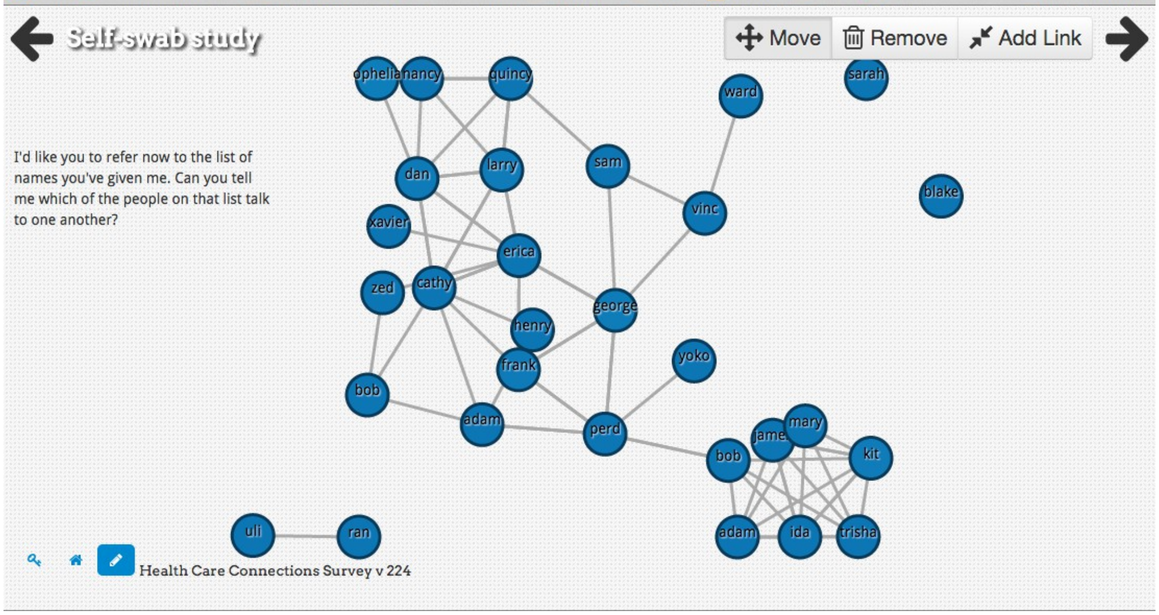

Supplement: Multimedia Appendix 1 [file resprot_v6i6e124_app1.pdf]
