# Supplementary figures and images for: An Interactive, Mobile-Based Tool for Personal Social Network Data Collection and Visualization Among a Geographically Isolated and Socioeconomically Disadvantaged Population: Early-Stage Feasibility Study With Qualitative User Feedback
Source: JMIR Res Protoc. 2017 Jun 22;6(6):e124. doi: 10.2196/resprot.6927 (PMC5500782; doi:10.2196/resprot.6927)

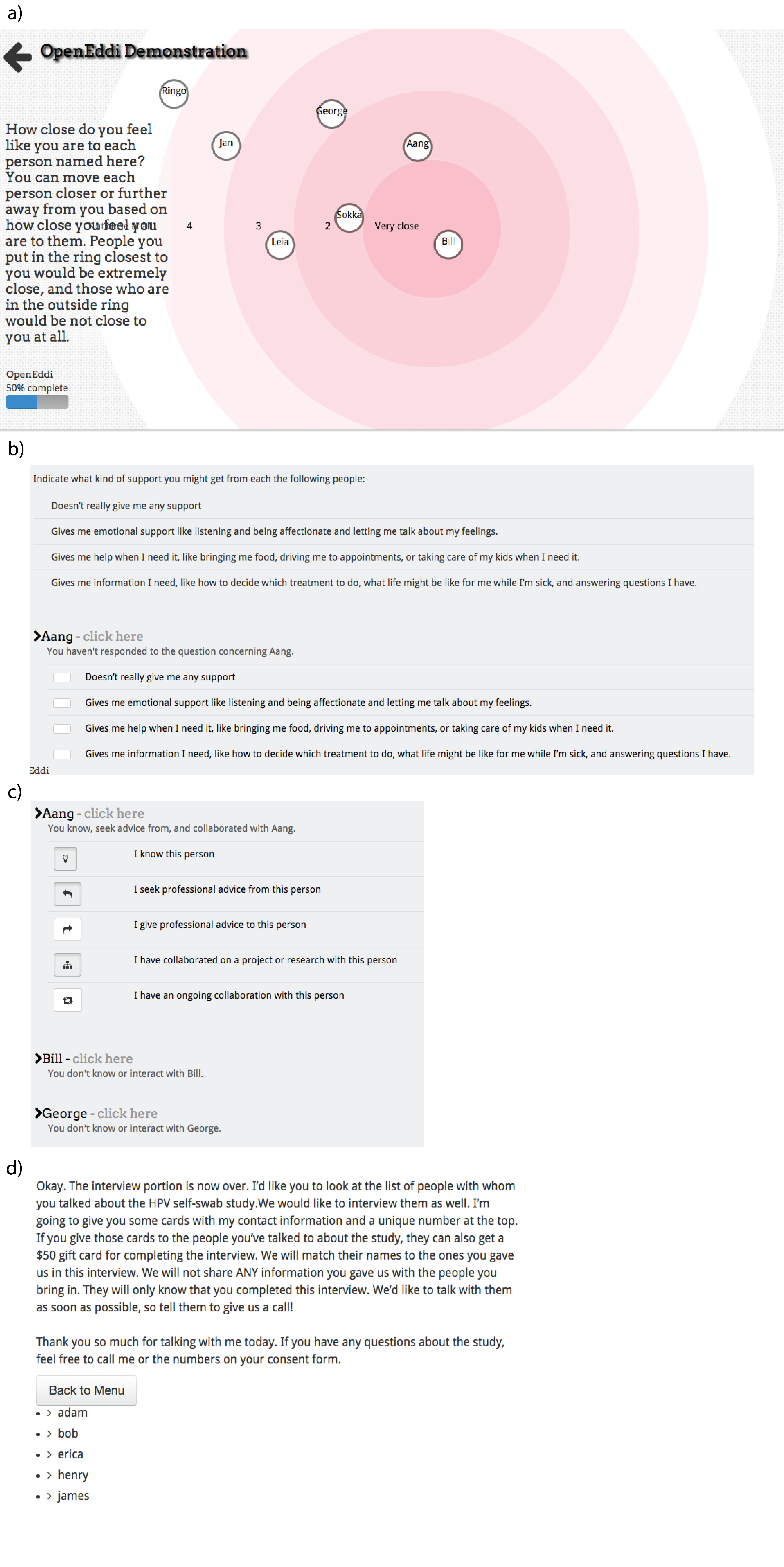

Supplement: Multimedia Appendix 3 [file resprot_v6i6e124_app3.png]
